# Neutron transport calculation for the BEAVRS core based on the LSTM neural network

**DOI:** 10.1038/s41598-023-41543-1

**Published:** 2023-09-06

**Authors:** Changan Ren, Li He, Jichong Lei, Jie Liu, Guocai Huang, Kekun Gao, Hongyu Qu, Yiqin Zhang, Wei Li, Xiaohua Yang, Tao Yu

**Affiliations:** 1 School of Nuclear Science and Technology, University of South China, Hengyang, Hunan China; 2 School of Computer Science and Engineering, Hunan Institute of Technology, Hengyang, Hunan China; 3 School of Computing/Software, University of South China, Hengyang, Hunan China; 4 Nuclear and Radiation Safety Center, MEE, Beijing, China

**Keywords:** Power stations, Engineering, Applied mathematics, Computational science

## Abstract

With the rapid development of computer technology, artificial intelligence and big data technology have undergone a qualitative leap, permeating into various industries. In order to fully harness the role of artificial intelligence in the field of nuclear engineering, we propose to use the LSTM algorithm in deep learning to model the BEAVRS (Benchmark for Evaluation And Validation of Reactor Simulations) core first cycle loading. The BEAVRS core is simulated by DRAGON and DONJON, the training set and the test set are arranged in a sequential fashion according to the evolution of time, and the LSTM model is constructed by changing a number of hyperparameters. In addition to this, the training set and the test set are retained in a chronological order that is different from one another throughout the whole process. Additionally, there is a significant pattern that is followed when subsetting both the training set and the test set. This pattern applies to both sets. The steps in this design are very carefully arranged. The findings of the experiments suggest that the model can be altered by making use of the appropriate hyperparameters in such a way as to bring the maximum error of the effective multiplication factor keff prediction of the core within 2.5 pcm (10^–5^), and the average error within 0.5266 pcm, which validated the successful application of machine learning to transport equations.

## Introduction

The main task of the reactor physics analysis is to simulate the various nuclear processes in the core to give the key parameters related to neutron dynamics in the nuclear reactor^1,2^. The "four-factor model" and "six-factor model" played an important role in the early physical analysis of reactors.

The solution of the neutron transport equation in differential-integral form requires the decoupling and discretization of the variables, and the current methods for angular discretization include the spherical harmonics method (PN)^3^ and the discrete ordinates method (SN)^4^. The discrete ordinates method uses individual angular directions instead of the entire angular space to discretize the angular variables and obtain the neutron balance equation in the specified direction. With the rapid development of computing and technology, the method of characteristic lines (MOC)^5^ and the Monte Carlo^6^ transport calculation methods have also been developed significantly. The characteristic line method converts the neutron transport equation into a one-dimensional neutron transport equation by using a series of mutually parallel characteristic lines covering the entire solution region. The Monte Carlo method, on the other hand, involves generating different particle initial positions, energies, and emission angles, as well as simulating various processes within the medium (such as production, collision, disappearance, and termination, etc.). The information obtained is then subjected to mathematical and statistical analysis. By utilizing a large number of random numbers, a stochastic model is constructed, and the model is solved using physical processes, the key to its solution lies in the reasonable use of a large number of random processes to simulate the random motion of neutrons in various media, and to solve the contribution of this motion process to a certain physical quantity.

## LSTM neural network

High-speed research in neural networks, deep learning algorithms, and reinforcement learning is largely driving the AI revolution. These sophisticated algorithms can handle very complex machine learning tasks that are characterized by nonlinear relationships and interactions between features with a large number of inputs. Long Short-Term Memory (LSTM), first proposed in 1997^7–9^, is a neural network specifically proposed to solve the problem of long-term dependence in general recurrent neural networks.

**Figure 1:**
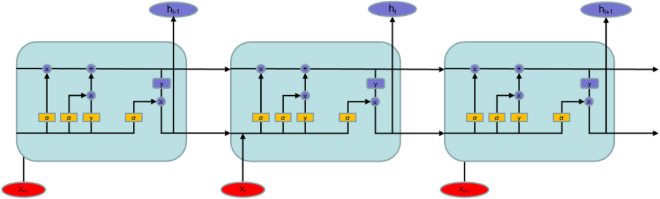
LSTM cell structure diagram.

The cell structure of the LSTM model is shown in Fig. 1, and its calculation formula is shown in Eqs. (1)–(5)^10^ are shown.

1 $$ i_{t} = \sigma \left( {\sum {W_{xi} x_{t} + } \sum {W_{hi} x_{t - 1} + } \sum {W_{ci} x_{t - 1} + } b_{i} } \right) $$

2 $$ f_{t} = \sigma \left( {\sum {W_{xf} x_{t} + } \sum {W_{hf} x_{t - 1} + } \sum {W_{cf} x_{t - 1} + } b_{f} } \right) $$

3 $$ o_{t} = \sigma \left( {\sum {W_{xo} x_{t} + } \sum {W_{ho} x_{t - 1} + } \sum {W_{co} x_{t - 1} + } b_{o} } \right) $$

4 $$ \tilde{c}_{t} = {\text{f}}_{{\text{t}}} c_{t - 1} + i_{t} \tanh \left( {\sum {W_{xc} x_{t} + } \sum {W_{hc} x_{t - 1} + } b_{c} } \right) $$

5 $$ {\text{h}}_{t} = o_{t} \tanh (\tilde{c}_{t} ) $$

At time $t$, the input vector is represented by $x_{t}$. The weight bias term is denoted by $b$. $\sigma$ refers to the activation function. The cell structure state values at moments $t$ and $t - 1$ are represented by $\tilde{c}_{t}$ and $c_{t - 1}$, respectively. The hyperbolic tangent function,$\tanh$, is used as the activation function. The input gate is denoted by ${\text{i}}_{t}$, and it corresponds to the weights $W_{xi}$, $W_{hi}$, and $W_{ci}$. The forgetting gate is represented by ${\text{f}}_{t}$, with corresponding weights $W_{xf}$, $W_{hf}$, and $W_{cf}$. Similarly, the output gate is denoted by ${\text{o}}_{t}$, with corresponding weights $W_{of}$, $W_{of}$, and $W_{of}$. The cell output value at time t is represented by ${\text{h}}_{t}$.

## BEAVRS core introduction

**Figure 2:**
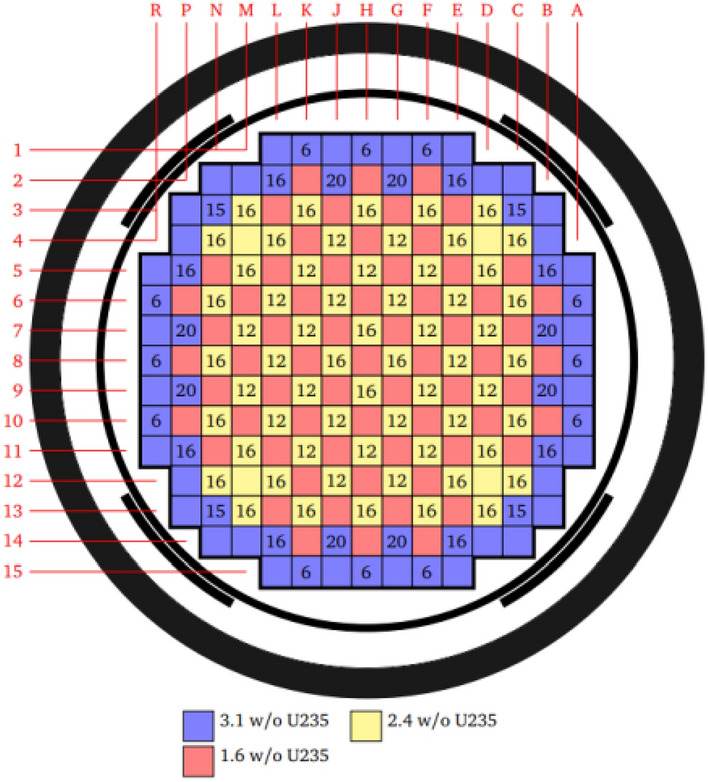
Core arrangement diagram.

The BEAVRS model^11^ is derived from a real pressurized water reactor from Westinghouse. The basic structure and assembly enrichment distribution are shown in Fig. 2.

The core contains 193 fuel assemblies, and the fuel rods in the assemblies are arranged in a 17 × 17 lattice. Each assembly contains 264 fuel rods, and one instrument tube is installed at the center of the core. One instrument tube is installed in the center for in-stack measurement, and 24 guide tubes are installed around the center. The fuel assembly parameters are shown in Table 1.

**Table 1:** Fuel assembly parameters.

Parameter	Value	Parameter	Value
No. fuel assemblies	193	No. guide tube	24
Fuel assembly lattice pitch/cm	21.50364	Guide tube ID (above dashpot)/cm	1.12268
Pin lattice configuration	17 × 17	Guide tube OD (above dashpot)/cm	1.20396
Pin lattice pitch/cm	1.25984	Guide tube ID (at dashpot)/cm	1.00838
Outer diameter of fuel pin/cm	0.9144	Guide tube OD (at dashpot)/cm	1.09220
Fuel rod diameter/cm	0.78436	Guide tube material	Zircaloy
Heavy metal loading (MT*)	0.42383	Cladding thickness/cm	0.05715
Fuel rod material	UO_2_	No. instrument tube	1
Cladding material	Zircaloy	Instrument tube OD/cm	1.20396
Top/bottom grid spacer material	Inconel 718	Instrument tube ID (air)/cm	0.87376

*MT* metric ton, *ID* inner diameter, *OD* outer diameter.

**Figure 3:**
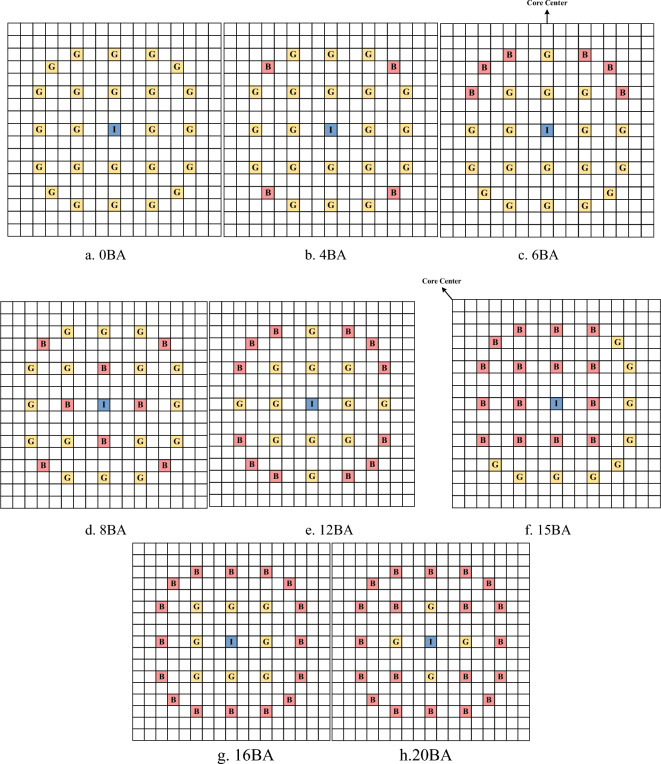
Burnable absorber assembly model (B: burnable absorber rod, G: guide tube, I: instrument tube).

Table 2 gives the basic parameters of the assemblies containing burnable absorber rods. Figure 3 gives the arrangement of combustible absorber in the assembly.

**Table 2:** Parameters of burnable absorber rods.

Parameter	Burnable absorber
Burnable absorber material	12.5% borosilicate glass
burnable absorber rod OD/cm	0.96774
Density/g cm^−3^	2.26
Cladding material	SS304
Cladding outer thickness/cm	0.04699
Cladding inner thickness/cm	0.01651
Air gap material	Helium
Air gap outer thickness/cm	0.01016
Air gap outer thickness/cm	0.01079

Based on the BEAVRS benchmark core description, the corresponding two-dimensional core fuel burnup calculation model is established; the lattice calculation adopts the multi-group two-dimensional transport theory for fuel burnup calculation, and two group cross-sections are obtained for each assembly type under each fuel type. The lattice calculations are performed using the DRAGON code^12,13^.

The DONJON code is used to calculate the core fuel burnup^14^. The DRAGON4.1 and DONJON4.1 were reactor numerical analysis programs developed by the Polytechnic University of Montreal, Canada. Among them, DRAGON4.1 was designed around the solution of the neutron transport equation. DRAGON4.1 is lattice code that contains several computational modules. The main computational modules were the fine-group micro-section database processing module LIB, the geometric feature description module GEO, the spatial discretization module based on the collision probability modules SYBILT, EXCELT, and NXT,, discrete coordinate module SNT, characteristic line module MOC; resonance processing module includes resonance processing module based on the equivalence principle. The resonance processing module includes the resonance processing module SHI based on the equivalence principle, and the subgroup resonance module USS; the transport equation solving module FLU, EDI, EVO, etc. The above modules were implemented in the software package through the program GAN.The above modules were connected together by the program GAN within the package, and the data were exchanged between the modules through a well-defined data structure. In the modeling process, the power was chosen to be constant and the boron concentration was kept constant.

**Figure 4:**
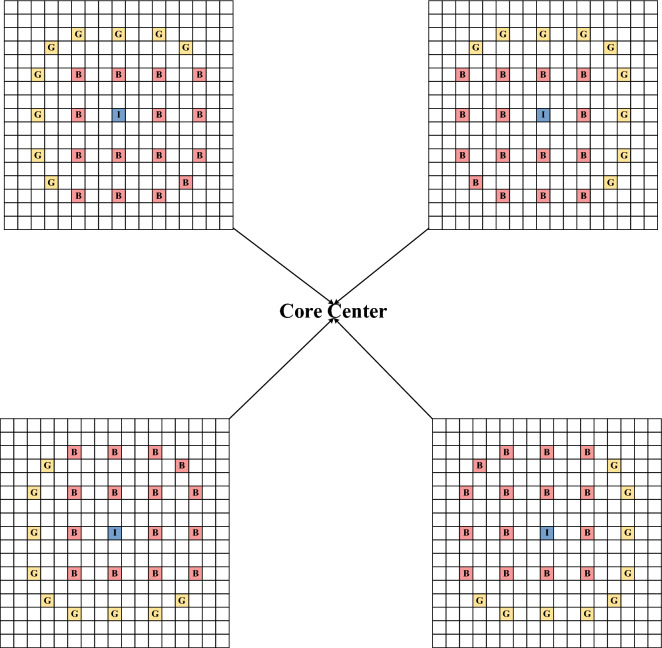
Four different assemblies with 15 absorber fuel rods arrangement methods.

As shown in the Fig. 3, 17 × 17 fuel rods modeling method was chosen in the lattice calculation,, and there were 15 assembly forms according to the distribution of enrichment and absorber, including 1.6% enrichment without absorber, 2.4% enrichment without absorber, 2.4% enrichment with 12 absorber fuel rods, 2.4% enrichment with 12 absorber fuel rods, 3.1% enrichment No combustible absorber, etc. The 3.1% enrichment assembly with 6 absorber fuel rods and the 3.1% enrichment assembly with 15 absorber fuel rods were divided into 4 different assemblies due to their asymmetry as shown in Fig. 4. In the calculation of the lattice, the fuel rod pincell, the guide tube pincell, the burnable absorber pincell and the instrumentation guide tube pincell were filled at different locations according to each assembly, and their specific geometric material structures were shown in the reference^15,16^. In the assembly modeling process, the boundary conditions were selected as reflection, and the transport equations were solved by the collision probability method. In the selection of the multi-group interface library, the 69-group cross-section library in IAEA's WIMSD4 database is selected, and the DRAGON-readable file format was generated by the NJOY program, and the 2-group homogenized few-group interface is generated by DRAGON for DONJON to read. In the DONJON modeling, 17 × 17 core modeling was selected, and the corresponding assemblies were filled in the middle according to the BEAVRS core arrangement, and the water reflection layers were filled in the periphery of the assemblies. DONJON performs core physics calculations by reading transport cross-sections of various assembies obtained through DRAGON and employing a coarse mesh finite difference method.

## LSTM modeling process

**Figure 5:**
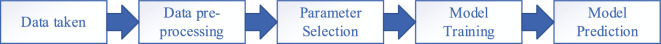
Flow chart of LSTM-based core effective multiplication *k*_*eff*_ prediction method.

The LSTM neural network was used to predict the BEAVRS core effective multiplication factor *k*_*eff*_, and different hyperparameters were taken to set up the prediction model^17–20^, and the specific process method is shown in Fig. 5.

### Data pre-processing

The effective multiplication factor *k*_*eff*_ was calculated by DRAGON/DONJON over 0–300 days at maximum power with a sampling frequency of one day. Because of the significant difference in the range of values of different feature quantities, the linear normalization^17^ method (i.e., the maximum normalization method) is used to normalize the feature significant quantities to achieve better model accuracy. The formula is shown in Eq. (6), where *x* is the initial feature value *k*_*eff*_, *x*_*max*_ is the feature maximum, *x*_*min*_ is the feature minimum, and x^*^ is the processed feature value^20^.

6 $$ x^{*} = \frac{{x - x_{\min } }}{{x_{\max } - x_{\min } }} $$

### Model training

In this study, the loss function used to train the model is the mean squared error (MSE), which is the proportion of the square difference between the predicted and actual values to the number of samples. Let the sample size be n, the anticipated k-effective value be y*, and the actual k-effective value is y. The formula for MSE is given in Eq. (7), from which it can be deduced that the lower the MSE, the less the error and the greater the prediction effect. The model's accuracy is determined by comparing the absolute error (y* − y) of the predicted k-effective value to the actual value.

7 $$ MSE = \frac{1}{n}\sum\limits_{i = 1}^{n} {\left( {y_{i}^{*} - y_{i} } \right)^{2} } $$

In the training set, the prediction model is formed using the processed data and the hyperparameter settings indicated in Table 3. The trained model is used to test the test set according to the processes outlined below: based on the training set, the time steps are combined in order of 1–10 (interval of 1) depending on the performance of the computer used, the number of hidden neurons in the LSTM layer [4, 8, 16, 32], the model regularization coefficient 0.001–0.01 (interval of 0.001), the optimizer The model regularization coefficients are 0.001–0.01 (interval is 0.001), the optimizers are selected [adam, RMSProp, Adagrad, Adadelta], and the appropriate number of iterations epoch, batch size batch, callback function callbacks, and dropout rate dropout are selected.

**Table 3:** Model hyperparameter settings.

Parameter name	Set value
time_step	10–1
num_units	32, 16, 8 , 4
activation	tanh, sigmoid, relu
regularizer	0.1–0.0001
optimizer	Adadelta, Adagrad, RMSProp, adam
epoch	100
batch	5
dropout	0.3

**Figure 6:**
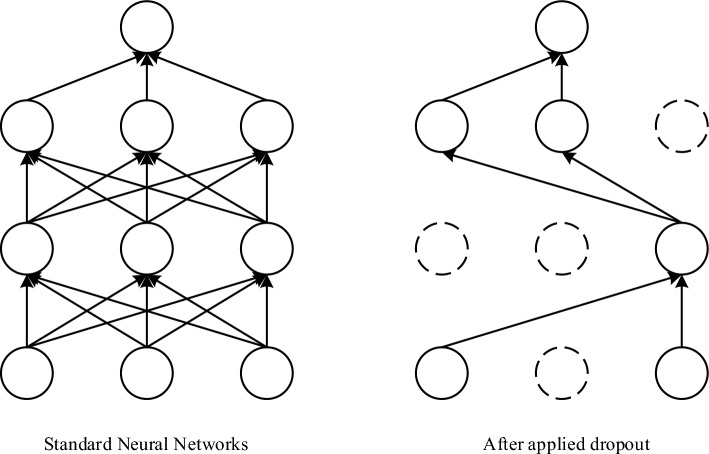
Dropout mechanism diagram.

It uses the L2 regularization factor in conjunction with the dropout layer to minimize model overfitting. Based on Occam's razor^21^, if anything has two explanations, the most probable true explanation is the one with the fewest assumptions, i.e., the most straightforward answer. Given certain training data and network design, the data may be explained by several weight values (i.e., multiple models). Complex models are more susceptible to overfitting than simple ones. Simple models are those that have fewer parameters. By lowering the complexity of the model by restricting the model weights to smaller values, the weight value distribution becomes more regular. This technique is referred to as weight regularization, which is accomplished by adding the cost associated with bigger weight values to the network loss function, adding L2 regularization factor i.e. the extra cost is proportional to the square of the weight coefficient (L2 norm of the weights) as indicated in Eq. (8), where *λ* is the regularization parameter, *E*_*in*_ is the training sample error without the regularization factor, and *L* is the loss function. Dropout^22^ refers to the deep learning training process. For the neural network training unit, it is eliminated from the network based on a given probability, for stochastic gradient descent. Figure 6 depicts the process of action, which prevents model overfitting by randomly deleting neurons.

8 $$ L = E_{{{\text{in}}}} + \lambda \sum\limits_{j} {w_{j}^{2} } $$

In machine learning, several optimization techniques^23,24^ are used to find the best model solution. In contrast to RMSProp, where the absence of correction factors may result in highly biased second-order moment estimates at the beginning of training, Adam contains bias corrections that account for the first-order moments (momentum terms) initialized from the origin and the (non-central) second-order moment estimations.

### Analysis of results

**Figure 7:**
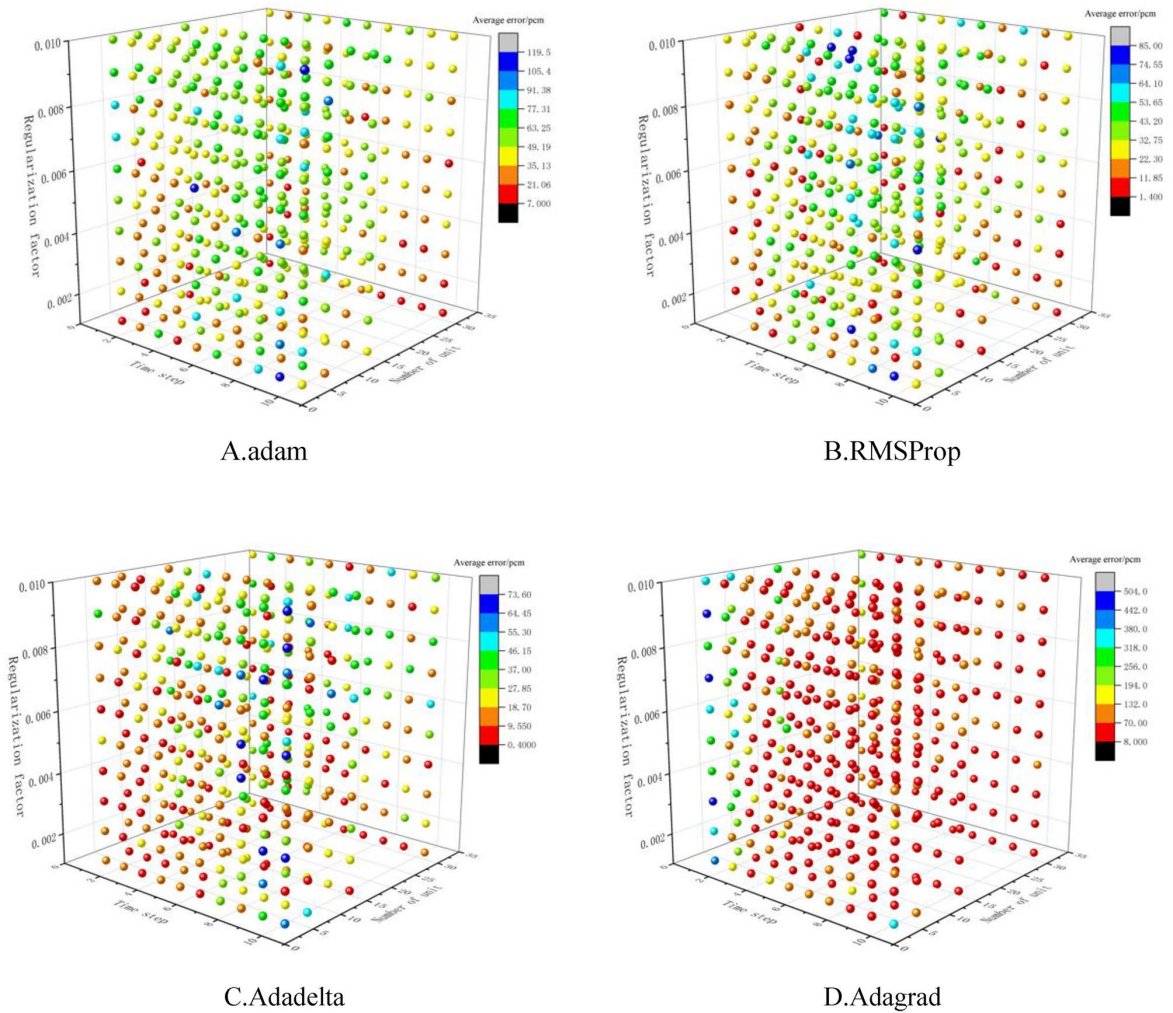
Error map of LSTM model.

The LSTM algorithm time steps were set to 1–10; the number of neural units was 4, 8, 16, and 32; the regularization coefficients were 0.001–0.01 and the optimizers were adam, RMSProp, Adagrad, and Adadelta respectively to model the first 65% of the data set, build a total of 1600 LSTM algorithm models for the next 35% of the data set do predictions and compare the errors, and the absolute error between the predicted and true values is used as the evaluation index, and the results are shown in Fig. 7.

It can be learned from Fig. 7 that for the problem of effective core multiplication factor *k*_*eff*_, the Adadelta-based LSTM algorithm model has the best prediction, followed by RMSProp and adam, and Adagrad has the worst prediction; for RMSProp, Adagrad, and Adadelta optimizers, the average error increases and then decreases as the regularization factor increases. the mean error increases then decrease, and then increases for the adam optimizer, while for the adam optimizer, it increases with the mean error, as shown in Table 4.

**Table 4:** Mean error variation with parameters.

Error variation	Time step	Number of units	Regularization factor
adam	Time step increases, error increases	As the number of units grows, the error drops and then increases	The regularization factor grows along with the inaccuracy
RMSProp	Time step increases, error increases	As the quantity of units grows, so does the margin of error	The regularization factor grows as the error first increases, then drops, and then increases again
Adadelta	Time step increases, error increases	As the number of units grows, the error drops and then increases	The regularization factor grows as the error first increases, then drops, and then increases again
Adagrad	Time step increases, error decreases	As the number of units grows, the error drops and then increases	The regularization factor grows as the error first increases, then drops, and then increases again

**Figure 8:**
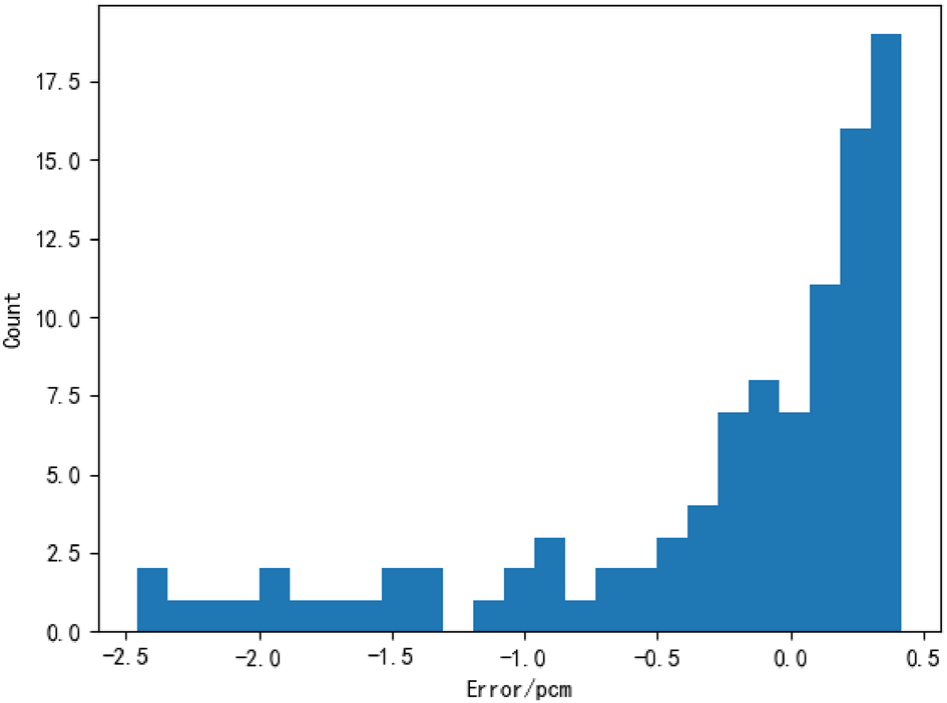
Error statistics chart.

By counting the 1600 models, a total of 138 models had an average error of less than 10 pcm, and the 10 models with the smallest average error were counted, as shown in Table 5. The model with the smallest average error (i.e., the time step of 3, number of cells of 16, regularization factor of 0.003, and optimizer selection of Adadelta) was subjected to error statistics, and the statistical results are shown in Fig. 8.

**Table 5.** Model error decimal table.

Serial number	Time step	Number of units	Regularization factor	Optimizer	Average error/pcm
1	3	16	0.003	Adadelta	0.5266
2	1	16	0.001	Adadelta	0.9370
3	2	4	0.002	Adadelta	0.9514
4	1	4	0.001	RMSProp	0.9742
5	1	8	0.001	Adadelta	1.1812
6	2	8	0.004	Adadelta	1.2292
7	1	16	0.001	RMSProp	1.5629
8	2	8	0.005	Adadelta	1.7697
9	2	8	0.001	Adadelta	1.7911
10	1	8	0.002	RMSProp	1.9308

## Conclusion

This paper focuses on exploring the feasibility of the LSTM (Long Short-Term Memory) algorithm in deep learning for effective multiplication factor *k*_*eff*_ prediction at the core level, modeled by BEAVRS (Benchmark for Evaluation And Validation of Reactor Simulations) core first cycle loading with *k*_*eff*_ of operating at full power for 0–300 days was used as the study subject. The first 65% of the dataset is the training and validation set, and the last 35% of the dataset is the prediction target. The training and alignment results of the physical parameters of the assemblies were obtained using the DRAGON4.1 and DONJON4.1 codes, and the LSTM algorithm in deep learning was applied. By adjusting the number of LSTM cells, L2 regularization parameters, optimizer type, and other parameter coefficients in the algorithm. The results showed that the absolute error of the predicted core effective multiplication factor *k*_*eff*_ could be made within 2 pcm by adjusting the appropriate parameters, which validated the successful application of machine learning to transport equations. In the future, we plan to fully leverage the advantages of big data to establish a unified model across multiple operating cycles or different physical models.

## Data Availability

The datasets generated during and/or analysed during the current study are available from the corresponding author on reasonable request.
